# “I have to do twice as well” – managing everyday racism in a Swedish medical school

**DOI:** 10.1186/s12909-022-03262-5

**Published:** 2022-04-01

**Authors:** Emelie Kristoffersson, Katarina Hamberg

**Affiliations:** 1 Department of Public Health and Clinical Medicine, Family Medicine, Umeå University, 901 87 Umeå, Sweden; 2 Umeå Centre for Gender Studies, Umeå University, 901 87 Umeå, Sweden

**Keywords:** Medical education, Interviews, Grounded theory, Everyday racism, Racial microaggressions

## Abstract

**Background:**

Mounting evidence suggests that medical students from cultural/ethnic minority backgrounds face recurring and more or less subtle racist oppression, i.e., everyday racism. Insights into how they handle these inequalities, though, are scarce – especially in a Swedish context. In this interview study we therefore explored and analyzed the strategies used by racialized minority medical students to manage episodes of everyday racism – and their underlying motives and considerations.

**Methods:**

Individual interviews were carried out with 15 medical students (8 women, 7 men) who self-identified as having ethnic- or cultural minority backgrounds. Inspired by constructivist grounded theory, data were collected and analyzed simultaneously.

**Results:**

Participants strove to retain their sense of self as active students and professional future physicians – as opposed to passive and problematic ‘Others’. Based on this endeavor, they tried to manage the threat of constraining stereotypes and exclusion. Due to the power relations in medical education and clinical placement settings as well as racialized students’ experience of lacking both credibility and support from bystanders, few dared to speak up or report negative treatment. Instead, they sought to avoid racism by withdrawing socially and seeking safe spaces. Or, they attempted to adopt a professional persona that was resistant to racial slights. Lastly, they tried to demonstrate their capability or conform to the majority culture, in attempts to refute stereotypes.

**Conclusions:**

Racism is not caused by the exposed individuals’ own ways of being or acting. Therefore, behavioral changes on the part of minority students will not relieve them from discrimination. Rather, strategies such as adaptation and avoidance run the risk of re-inscribing the white majority as the norm for a medical student. However, as long as racialized minority students stand alone it is difficult for them to act in any other way. To dismantle racism in medical education, this study indicates that anti-racist policies and routines for handling discrimination are insufficient. School management should also acknowledge racially minoritized students’ experiences and insights about racist practices, provide students and supervisors with a structural account of racism, as well as organize training in possible ways to act as a bystander to support victims of racism, and create a safer working environment for all.

## Introduction

Inequalities and discrimination related to culture/ethnicity/race is a persistent problem worldwide. Neither physicians nor medical students are spared these injustices [1**–**3]. The disproportionate number of cultural/ethnic minority health care workers and patients dying in the current Covid-19 pandemic, in e.g., the US, UK, and Sweden has heated the already ongoing debate about racism and structural discrimination in medicine following the Black Lives Matters movement [4**–**6]. Researchers, physicians, and students have called on the medical community to take actions against structural racism within their own institutions [6–11].

Recurring and subtle forms of racism constitute an often overlooked obstacle to the success and prosperity of medical students from minority backgrounds [3, 12–16]. Invalidations of this kind have been outlined as everyday racism and/or racial microaggressions i.e., intentional or unintentional commonplace indignities that convey hostile or derogatory slights and insults based on perceived differences in cultural/ethnic background [17, 18]. Such practices sustain a privileging of white majority over racialized minority students [13, 15], and can have deleterious effects on minority students’ mental and physical health [12, 18].

Research shows that everyday racism and/or racial microaggressions are difficult for victims to handle since these practices often are normalized, and because they are given meaning through their repetition [17, 18]. To date, however, only a few studies have discussed how racialized minority medical students cope with the stressors of racism [13, 15], and even fewer have explicitly examined this subject **–** and as far as we know there are no previous Swedish studies in the field. Furthermore, to deal with stereotypes imposed on them, people are forced to consider the rules, norms, societal discourses, and power relations that characterize the context in which they find themselves [17, 19, 20]. Therefore, a study taking into consideration the historical and contemporary relations in Swedish medical schools is warranted.

Tolerance, social justice, and equality characterize the Swedish self-image, and racism and discrimination are considered to be minimal [21]. However, being perceived as an ‘Immigrant’ – which often can be equated with being seen as racialized/non-white – has proven to be a basis for discrimination in terms of inferior health, lower life expectancy, housing standards, education, and employability [22–24]. In a previous interview study, we showed that while white minority students perceived the study climate as friendly and including, racialized minority students faced everyday racism and/or racial microaggressions [14]. They regularly encountered elusive adverse treatment from supervisors, peers, staff, and patients. A dearth of support from bystanders was a common aspect of their stories. These experiences pointed them out as ‘Others’ and made them feel less worthy as medical students.

In the present study, the aim is to continue our work by analyzing the racialized minority medical students’ strategies to manage episodes of everyday racism during their education – and their underlying motives and considerations. This study offers a deeper understanding of racialized students’ situation and indicates how medical education can work to counter discrimination.

Consistent with emergent design, the analysis of the empirical material advised our choice of theory [25]. We adopt a structural account of racism, perceiving it as a process involving an uninterrupted, often unconscious, exercise of power that is based on the privilege of whiteness and the taken-for-granted superiority of Western culture [17, 26, 27]. To understand how these processes operate in everyday life at the clinic we draw on Essed’s (1991) theory of ‘everyday racism’ and Sue’s (2010) theory on ‘racial microaggressions’ as described above [17, 18]. Culture, ethnicity, and race, in turn, are understood as social constructs where people are set apart from each other based on e.g., language, religion, and ways to dress, and not as innate characteristics [28].

## Method

### Research design

In this interview study, we deployed a constructivist grounded theory (GT) approach [25]. Individual interviews were conducted to allow exploration of the potentially sensitive topic of social exclusion. Constructivist GT was selected because of its emphasis on meaning being (re)created in interaction and people acting on the meaning they ascribe to a particular situation.

### Setting

This study was conducted at Umeå University in Sweden. In Sweden, the undergraduate medical curriculum encompasses 5,5 years (11 semesters), and the last 6 semesters contain clinical training at hospitals and health care centers. Undergraduate education is followed by 18–24 months of internship, after which one can apply for a license to practice medicine and for a position as a resident.

At Umeå’s Medical School, each class includes approximately 100 students. The latest figures suggest that less than 10% of the students are foreign-born or have two foreign-born parents [29]. The corresponding figure for all medical students in Sweden is 18% [30].

Swedish Universities are legally bound to work against discrimination related to e.g., sex, ethnicity, religion, or other beliefs [31]. Consequently, an Equal Treatment Plan, outlining how equal rights are to be promoted, how to inhibit discrimination, and a “zero tolerance” policy for discrimination, harassment, and abusive treatment is adopted within Umeå University [32, 33]. There are no rules about including education about discrimination and racism in the medical curriculum in Sweden. Still, in the Professional Development course, running like a thread throughout medical education in Umea, there are a few seminars focusing on cultural awareness and racism.

### Participants

Selection criteria for inclusion in the original study were belonging to an ethnic, cultural, or linguistic minority – as determined by the students themselves. In the Swedish context, minorities constitute a heterogeneous group and people who have immigrated rarely define themselves in term of their ethnicity [22, 28, 34]. Therefore, we stressed a broad definition of minority with examples like “having moved to Sweden as an adult, child, or being born here with parents born abroad” or “having a first language other than Swedish”.

Eighteen participants were recruited through e-mail and/or information adjacent to lectures directed to all students registered in clinical semesters (*N* = 8), a direct invitation from one of the researchers (*N* = 1), and snowball sampling (*N* = 9). One interview was conducted with each participant. Three of the participants belonged to a linguistic minority, were light skinned, and born in Northern European Countries. They reported no experiences of racism. Due to the aim of the present study, we only included the 15 interviews conducted with students who described being racially minoritized (8 women, 7 men) in the analyses. They were between 22 and 38 years old. Ten were either born in, or had parents born in countries in Middle East and Asia, one in Europe, and four in African countries.

### Data collection

Between 2016 and 2018, interviews were carried out by the first author (at that time a medical school graduate) and by a medical student (later medical intern) employed as a research assistant – both white women.

Interviews followed a topic guide exploring participants’ own medical school experiences, interactions with co-students, supervisors, staff, and patients as well as other situations where their origin became important in a positive or negative way; and how participants dealt with these experiences. Follow-up questions were posed to encourage participants to provide examples and clarify details. Efforts were made to establish a safe atmosphere for sharing personal experiences [25, 35].

Interviews were 57 to 125 min long, digitally recorded, and transcribed verbatim. Before the interviews, interviewees completed a brief demographic questionnaire.

### Analysis

Data collection and analysis were iterative, allowing us to let emerging insights inform the consecutive interviews to saturate the emerging categories [25]. We stopped interviewing when we assessed that saturation was reached, i.e., that on a higher abstraction level no new insights emerged.

The analysis was inspired by the constant comparison technique of GT and by our theoretical framework. We (EK, and KH) individually read and coded the transcripts. Each interview was also condensed into a case narrative, reflecting the previous coding. Then the codes were compared, discussed in relation to the theoretical framework, and grouped into preliminary categories that included the content of participants’ experiences. At this stage we decided to divide the analysis into two parts; one focusing on various experiences of everyday racism (number 14 in the publication list) and the present study focusing on affected students’ way to handle the discrimination they encountered. For the present analysis EK re-scrutinized the codes, categories, and case narratives, read the 15 interviews again and interpreted the material by means of focused coding on ways to react to and manage the problematic experiences. The preliminary categories created were compared and refined, and a core category, “Always on the alert: managing the threats of racism”, was identified. Here, a core category refers to a category that embraces all the other categories. Lastly, EK reviewed the 15 interviews again, and KH reassessed a sample of four interviews to confirm that the categories were grounded in the data.

Epistemologically, we acknowledge that the data are co-constructed between interviewer and interviewees. During the entire process, EK and KH therefore maintained reflexivity through critical reflections and discussion about the assumed associations between the emerging categories, the theoretical framework, and their own roles in the research process [36].

### Ethics

Studying a marginalized group, like racialized minority students, always implies the risk of reifying them as a fixed category of ‘Others’ [37]. We handled this dilemma by striving to understand the individual participant’s perspective, posing follow-up questions, asking for concrete examples, and by searching for ‘negative cases’ to pick up differences.

To ensure confidentiality, detailed background information for participants has been withheld, and details about individual people emerging in the interviews have been deleted or modified. Participants were given oral and written information about the study and their written consent was obtained before the interviews.

The Regional Ethics Committee in Umeå granted ethical approval for the study: (Dnr 2016/446–31).

## Results

Participants generously shared and reflected upon how they navigated and negotiated adverse incidents involving supervisors, staff, patients, and co-students. A core category was established, grounded in five categories, describing how participants managed their situation (see Table 1). Below, the core category and categories are presented and illustrated with quotations from the participants. Each quote is marked with the participant’s gender and interview number (Man = M, Woman = W).

**Table 1:** Summary of the analysis in a core category underpinned by five categories

Core category	Categories
Always on the alert: managing the threats of racism	Speaking up and confronting
	Formal reporting
	Searching for safe places
	Creating emotional distance
	Neutralizing threatening stereotypes

### Always on the alert: managing the threats of racism

The core category summarizes the strategies through which participants tried to manage the threats of racism.

Recurrent experiences of constraining stereotypes and exclusion that placed them as ‘Others’ and threatened their success as students made participants self-aware and hyper-vigilant: “I’m kind of prepared all the time” (15W). Often, they grappled with whether they could or dared take immediate action: “If something is really racist, then I want to speak up. But I have to consider the situation. It’s not always possible” (5W). However, they also took many precautionary measures to prevent or mitigate the risk of provoking stereotypes. Depending on the context, they utilized various strategies to handle the threats of racism. The prevailing power relations in the educational- and clinical situations and the potential impact on their success as students were major considerations in this process.

While participants spent considerable time and energy navigating the risk of stereotypes and exclusion, they seemed reluctant to view themselves as victims. Rather, they wanted to be perceived as active students and professional physicians-to-be.

### Speaking up and confronting

All participants highlighted that speaking up and confronting were important ways to oppose racialized slights and resist peoples’ attempts to categorize and confine them to racial or cultural stereotypes. To directly mark against injustice involved a shift of fault and responsibility to the perpetrator – which was described as empowering. Active protests, however, constituted only rare exceptions.

Many incidents were considered minor and/or ambiguous, making them difficult to recognize for peers and tutors and deemed not worth complaining about. One student explained how the complexity of such incidents combined with racialized students lacking credibility held him back:

People can just protect themselves. Because it is nothing concrete. They can defend themselves by saying: “No, I said nothing wrong. How can you accuse me of being a racist?” True! I can't do that, because they haven’t explicitly signed a paper where they say: “Hereby I certify that I have racist prejudices and oppress people because of it.” (4M)

Another reason given for not speaking up was fear of negative consequences. Interviewees thought they stood out in the crowd and speaking up they feared would magnify this: “I already felt different, and then I did not want to call attention by speaking up to the physician” (11W). Participants’ need to build trust with supervisors meant that their abilities to voice criticism without being discredited were circumscribed. Placing patients in an unfavorable situation by accusing them of racist utterances, in turn, interfered with their image of professional behavior. Maintaining a professional self-image in relation to supervisors and patients was thus accomplished by silently accepting racism.

In the rare instances when interviewees had chosen not to quietly accept tacit treatment or racist stereotypes, they seldom experienced any support or endorsement, rather, perpetrators and/or bystanders dismissed and invalidated the racial insult. Thus, in most situations they refrained from raising such issues: “I’ am a little afraid to end up in conflict with a teacher” (12M).

Participants sometimes attempted to object to injustice by exposing prejudices through seemingly innocent questions, forcing people to explain dubious statements:

An immigrant with stomach pain came in to the ER with an ambulance. Whereby the resident said: “They always do that.” I played a little stupid and said: “Okay, so this patient comes in often, and usually calls the ambulance?” Whereby the physician said: “Are you one of them who is going to call me a racist now?” (13W)

This quote highlights that even such factual comments opened up to scrutiny and made participants vulnerable to further aggressions. Knowing how frequently people reacted defensively when prejudices were called out made participants aware that protest might be damaging, triggering stereotypes depicting minorities as “oversensitive”.

There was also a striving to reclaim the position as ‘Other’ as “positive” and “normal” – and thus resist the image of the ‘Other’ as negative. Being recurrently confined to negative stereotypes some male students tried to convert them to a strength or at least something that they could use in certain situations. One stated that it could be nice to “play on prejudices”. If he did not want to be bothered, he explained, he just frowned, and then people let him be because they thought he looked frightening. This strategy, however, both indicates and reproduces racialized stereotypes.

### Formal reporting

To make use of recognized processes within the university for reporting discrimination directly to the course management was seen as important, but in reality difficult.

Participants were, on the one hand, content with the measures being undertaken to facilitate students’ reporting negative treatment through course evaluations: “There is like a zero tolerance for discrimination. It is a security to know that as a student you have your rights” (7W). On the other hand, even if these evaluations were anonymous, making complaints turned out to be practically impossible since participants feared they could still be identified due to the small number of racialized students: “It becomes quite obvious who has reported it” (13W). Another female student, who had experienced adverse treatment from “a distinguished physician”, said:

I would have wanted to go to the course management, or the course coordinator. But I don't know how. It's just me who hasn't dared to. So I think the error lies with me. I am the one who should change (15W)

The formal directives conveying to students that they can report discrimination seemingly make her blame herself for the continuous injustice. Fear of being questioned added further to participants’ hesitance to report discrimination: “To dare, one must be sure that one will not be questioned” (11W).

A few participants were confident that the course management would take measures if they found out about discrimination. Others believed supervisors could still get away with inappropriate behavior because of their important role in the health care organization.

Only one interviewee had reported poor treatment to the course management. He was astonished when the teacher in question listened to and confirmed his experience – and underlined how important this recognition had been for him.

### Searching for safe places

Trying to protect oneself from being subjected to racism and find contexts where one felt safe was important for participants.

They tried to escape and avoid specialties, clinics, and social settings where they had been – or feared they would be – ill-treated. At universities and hospitals with a larger proportion of racialized students and physicians, they imagined they would be treated fairly and “fit in better”. Thus, some dreamed about leaving Umeå altogether.

Experiences of mockery and scrutiny had also driven them to withdraw in educational contexts to avoid negative attention: “This fear that the doctor will be racist makes me feel I would rather not get in the way or don’t take up as much space” (10M).

However, to be successful, such measures require that only specific individuals are carriers of racism. As it turned out, friends also expressed preconceived opinions: “Some were people I was quite close to” (8W). After repeated incidents where he had to face biased beliefs from classmates, this participant gradually withdrew:

I have them at a safe distance where they can’t hurt me. Outside of school I meet none of my classmates. And that’s how I want it. I deny all invitations. When I let people come a little too close it usually gets wrong. If I put people in a position where they can hurt me, then it tends to happen too. So that’s why I keep my distance. (4M)

Isolation thus became a way to protect oneself from racism. This strategy, however, added to the segregation that placed participants in a subordinate position in the first place and entailed missing out on educational possibilities: “It means I can’t really use my full potential” (9M).

Moreover, participants strategically sought connection and affirmation to handle adverse experiences. Some turned to family and loved ones. Others underlined that it was paramount to have a network outside the family, e.g., minority classmates or friends, to receive validation. It was the shared experience of being constructed as ‘the Other’ that formed the basis for this mutual understanding and trust. Interactions with racialized students were described as synonymous with security, enabling participants to feel safe that one would not be questioned: “In my group of friends I am not judged for my language” (6W).

### Creating emotional distance

This category refers to cognitive strategies aimed at creating emotional distance to discriminatory experiences to alleviate their stressful effects.

Participants deliberately tried to reinterpret adverse experiences to protect themselves from pain: “I actively think that the person does not discriminate against me” (3M). Reinterpreting also worked to transform recurrent racist incidents into something they had the opportunity to influence, as described by this male participant:

I never want to believe it has to do with racism, so I give one million excuses instead. Because otherwise it will be so terrible to live. So I think it’s a self-defense mechanism for me that makes me survive one more day. […] Because it is difficult when it depends on something that I have just been born to, not something that I can influence. Because I don’t know what to do... What can I say? “Sorry, I’m black.” (4M)

Others indicated that they had learned to brace themselves and become impermeable to racial slights: “I’ve become immune; I don’t take it personally” (14W). Referring to their lack of opportunities to decide which images of them should be valid, they emphasized the importance of separating self-confidence from other people’s opinions: ” If I myself become the source of my self-confidence, of how I handle things, of how badly I feel when someone says something, then I become unwavering” (4M).

Another example of how emotional distance was established was to adopt a professional persona, e.g., to “think about what was be best for the patient”, be objective, rational, and not easily take offense, as explained by this female student:

I take on some sort of role: now I'm a professional. Obviously, you can be affected, get sad. But I think I can let it go quite quickly. I have the attitude that I am a caregiver. It's a bit like being a parent to someone. You are the adult, and then you do not take as much offence. If it’s my best friend, then I am much more sensitive. (5W)

The role of caregiver allowed her to uphold a positive self-image even in the presence of downgrading comments. This strategy, though, requires a condescending view of patients and frees them of responsibility for their actions.

In the end, keeping painful experiences at a distance was difficult, and some interviewees yielded to self-accusation when being unable to do so:

I perceive myself as weak. And let myself be impeded by other people. I believe that many with a foreign background have experienced the same things that I have, but if you are a little weak then I think you become inhibited. I have become so restrained because of everything that has happened. But I think if you are a little stronger and more confident, and don’t let anyone trample on you, then there’s no problem. (15W)

By suggesting that caring about peoples’ opinions is a sign of weakness in this way, the focus is transferred from the perpetrators and their inappropriate behavior onto the participants themselves and their (in) ability to handle such incidents.

### Neutralizing threatening stereotypes

This strategy implied a striving to be in control, to conform, and to adapt to societal expectations and discourses and become accepted by refuting stereotypes.

When facing rude and intrusive questions about e.g., their background or religion from supervisors, staff, and patients, or encountering patients’ skepticism of them participating in care, interviewees said that they felt it necessary to be patient and informative, providing comprehensive answers in order to sustain positive relations: “As an immigrant, you must first make sure that the patient is comfortable with you” (7W). Consequently, it seemed participants had to disregard their own needs to ameliorate the potential distress of white people. One student explained how he responded to questions about his background:

I’ve tried to respond to it by continuing to say: “I'm from [hometown]” until they ask: “Where are your parents from?” Then I always give in and say: “I am from [hometown] but the parents are from X-country.” And then they let go of the question immediately. (10M)

Experiences of being devalued, overlooked, and questioned compelled participants to put themselves forward and demonstrate their capability and knowledge: “It always feels like I have to prove myself a little extra because I’m not like everyone else (11W). They talked more during group work, posed many questions to teachers and supervisors, and carefully answered questions. Not because they wanted to excel or learned more by doing so, but because they felt they had to: “So that they see that I am a medical student. I speak Swedish. I’m just like everyone else” (11W). They hoped their efforts would protect them from negative preconceptions:

Sometimes I’m afraid that someone will judge me based on my appearance. I don’t think I would feel like that if I was a blonde. Because I look in a certain way, I have to do twice as well, so that no one will think: “It is that immigrant.” I do my best and it feels like it should protect me in some way against prejudice. (8W)

Some participants, though, asserted that the prospect of being discriminated against served as a driving force for improvements:

It motivates me. Immensely. I wouldn’t say that I feel that I have something to prove, because I don’t experience that. But it is still a driving force that ... it feels good to get other people to understand that, yes but: “He can do it too”. (1M)

Even though this student talks about discrimination in almost positive terms, as a driving force and motivator, it might still be argued that he needs those in power to acknowledge him as a worthy medical student. And that he is forced to work extra hard just to rise to the level of the white majority students.

In order not to trigger hostile stereotypes, participants also described behavioral changes aimed at conforming to perceived majority culture. For instance, being mindful of using correct Swedish, and avoid making jokes. One male student said: “I have changed behavior a lot, in order not to stand out too much” (12M). He underlined, though, that his adaptation did not constitute a real internalization of perceived majority norms. Like most interviewees, he displayed a resistance towards assimilation combined with a change of his outer persona to mimic desirable qualities: “You have a facade that you show others” (12M). Such adaptation, in turn, it might be argued, contributes to the normalization of cultural hierarchies.

## Discussion

We explored how racialized minority medical students handled incidents of everyday racism perpetrated by supervisors, staff, patients, and co-students. Participants managed the threat of constraining stereotypes and exclusion, while attempting to retain a sense of self as an active and professional physician-to-be – as opposed to a passive and problematic ‘Other’. Their modes of action were constrained by the educational- and clinical power hierarchy, minority students lacking credibility, a dearth of support from bystanders, and ideas about professional behavior. The strategies included: “speaking up and confronting”; “formal reporting”; “searching for safe places”; “creating emotional distance”; and “neutralizing threatening stereotypes”.

### Navigating implicit white norms on medical professionalism

By being seen as cultural and/or ethnic ‘Others’, our participants risked being discerned as missing qualities required of a good physician-to-be. This is in line with previous studies that have shown that assessment of academic excellence and professionalism is informed not only by official criteria, but also by whether or not one fits the norm of belonging to the white majority [2, 38, 39]. Therefore, it is not surprising that students’ strategies evolved around retaining their sense of being active and professional physicians-to-be. Furthermore, in line with previous studies participants seemed to confuse professionalism and professionalization, thinking that emotional detachment was a necessary component of professionalism [40]. Being offended by racial slights and/or speaking up was therefore viewed as lacking professionalism.

Important parallels can be drawn between the strategies used by our participants and those formulated and described by other scholars [17–19, 27, 41, 42]. All approaches carried both advantages and disadvantages. Acts of resistance could be liberating, but participants lacked credibility and speaking up made them vulnerable to accusations of being oversensitive troublemakers [17, 18]. Furthermore, they could not rely on support from bystanders. Hence, as seen in other studies racial slights were often perceived as not worth complaining about or reporting, especially in relations characterized by power differentials [13, 16, 18]. Resisting the downgrading of cultures other than “Swedish culture” by embracing otherness, in turn, meant that the interviewees identified with stereotypes created by the prevailing power order [17, 18, 42].

Standing alone and being unable to prove – or even protest against – discrimination, participants had to find other strategies for managing their situations. Searching for a safe place mitigated stress and meant having one’s experiences reaffirmed. However, as noted by other researchers it also entails a withdrawal from contexts students have the right to seize upon and makes them more isolated and vulnerable [15]. Creating emotional distance; a strategy commonly described in previous literature, helps keep the pain away, but means that the focus shifts from unfair events to the participants’ (in) ability to remain unaffected by them [18, 42]. Even if participants describe themselves as immune to racial slights, it remains something they have to handle. Furthermore, studies show that being continuously subjected to racial slights is psychologically stressful, resulting in e.g., frustration, anxiety, and anger [43]. Lastly, attempting to neutralize stereotypes by e.g., demonstrating one’s capability, or conforming to the majority culture could be rational in a competitive educational environment. However, interviewees’ chances to prove their capability are probably limited because in the eyes of the majority they will likely remain representatives of ‘the Other’ [19, 42].

Common to many of participants’ strategies is that they do not question the basic assumptions behind their marginalization, namely the division into essential groups based on culture/ethnicity [19]. Rather, strategies such as silence, adaptation, and avoidance run the risk of re-inscribing belonging to the white majority as representing the norm for a medical student. Concurrently, it means participants make themselves responsible for handling something that is not their fault, while perpetrators are deprived of responsibilities for their actions. But, when racialized minority students’ experiences of and knowledge about racism are not acknowledged and they are left without any support from bystanders, what are their possibilities for handling stereotypes and exclusion without reproducing their oppression?

This is the first study of its kind to be conducted among Swedish medical students. Although many of the strategies the participants used have been shown in previous research, our study also contributes with knowledge about the specific expressions that these strategies take in the hierarchical healthcare organization. In addition, our results are novel by illustrating how the strong association between professionalism and emotional detachment risks making it even more difficult to acknowledge that racism is a problem and make students even more prone to remain silent in the face of racism. And what is worse; racism risks being seen as something you as a professional should learn to put up with. Hence, this study also contributes important insights into the challenges and barriers that the work on counteracting racism may entail.

### Breaking the silence surrounding racism and discrimination

Anti-racist policies and routines for handling discrimination and harassment are important, but alone they are insufficient to prevent and manage racism and discrimination in medical school. Racialized minority students are in a subordinate position and too easily recognized to be expected to dare speak up and report. Also, being minority students, they often lack credibility, which is further undermined by spectators’ passivity. Lastly, because acts of everyday racism are often normalized making complaints about them is especially hard. Consequently, formal commitments to equality – such as those held in the medical school where this study was conducted – are at risk of becoming symbols of equality while inequalities remain [32, 33].

Countering racism in medical school will naturally require a long-term and comprehensive approach. On a structural level, it is for example important to continuously discuss and refine formal routines for reporting discrimination but other measures, such as increasing the proportion of teachers, tutors, and supervising physicians belonging to cultural minority groups are also needed. To break the silence surrounding racism and improve the situation for exposed students our results suggest that change must also occur in relationships and everyday practices at the clinics – with support from management. Students and not least teachers/supervisors should be assigned time for attending education on structural and everyday racism and/or racial microaggressions, which takes into account the power relations through which racism operates [17, 26, 27]. Otherwise, there is a risk that what our participants are exposed to will not be considered and/or identified as racism. Furthermore, witnesses to racial slights should be supported to move from passive bystanders to active allies to mark and establish that these practices are intolerable – a promising strategy according to recent studies [16, 44–46]. Addressing racism – both as a victim and as a bystander – naturally entail several challenges, not least the fear of retribution [16]. However, being an active bystander includes not only speaking up to perpetrators but also, for example, talking to other people who can address the incident and talking to and supporting the victim afterward [46]. Efforts to ameliorate these injustices should also include a discussion of what professionalism is – or should be – in order not to confuse professionalism with a stoic acceptance of discrimination and racism. To give these issues more focused attention, we propose dedicated theme days for both students and supervisors and mandatory teaching elements. Furthermore, finding new and alternative ways of reporting discrimination may be needed, as well as work on creating supportive networks.

### Methodological considerations, strengths, limitations & future research

Our committed sample of self-selected participants resulted in a rich material. Informants, however, may differ from those who abstained from participating. It is possible that those abstaining struggled to handle more grave experiences, but did not trust the researchers enough to participate in the study. Or they faced no challenges and thought they had nothing to contribute. The interviewees came from different backgrounds and their strategies varied, indicating that enrollment was broad, which offers credibility to the results.

Our data collection was performed prior to covid-19 and the attention that Black Lives Matter got during the first years of the pandemic. In Sweden, though, Black Lives Matter has not been discussed as a movement of much relevance for the Swedish society. Nevertheless, in the spring of 2021, an appeal from Swedish physicians and medical students against racism in Swedish health care was published in the media [47]. Later, it was revealed that caregivers throughout Sweden allow patients to choose to be cared for only by ethnic Swedes [48]. These events may have increased awareness of racism in medical education **–** and of the importance of addressing this problem. Consequently, had our data collection been performed now it is possible that our results would have been different.

That the interviewers shared the experience of being medical students with the participants may have enabled trust during the interviews. However, the interviewers were white, which means that participants may have avoided sharing information because of doubts that their perspective would be understood. Future research undertaken by researchers from racialized minority groups would therefore be valuable. Also, more research is needed on insidious forms of racism to inform strategies for effectively dismantling these inequalities. Lastly, in the work of counteracting racism, it will be important to ask for and listen to suggestions from students. This was not something that the participants in this study were asked about and can be seen as a limitation.

The study is also limited by its reliance on data from a single medical school. Nevertheless, similar strategies to those deployed by our participants have been seen and described in studies from other countries [13, 15]. Thus, we believe that our findings apply to other Swedish Universities and also other Western contexts. Still, future research should investigate experiences and reactions among racialized minority students in more culturally or ethnically diverse settings.

## Conclusions

Racialized minority medical students managed insidious racism including threats of constraining stereotypes, ‘Othering’ and exclusion, while at the same time maintaining a self-image of being competent medical students. Due to the clinical power hierarchy, racialized minority students lacking credibility and lack of support from bystanders, their strategies to counteract or avoid everyday racism were focused on adapting their own behavior or thinking – not on speaking up or formally complaining about discrimination.

However, constraining stereotypes and marginalization are not caused by exposed racialized students’ own ways of being and acting. Thus, strategies built upon behavioral modification will not free them from racism, nor will it grant them access to the desired position as active and professional physicians-to-be. Instead, strategies such as adaptation, silence, and avoidance risk entrenching belonging to the white majority as representing the norm for a medical student.

To counteract everyday racism in medical education, we suggest that medical school management embrace the knowledge that racialized minority students have about structural and subtle racism within our own institutions and impart this knowledge to all students and supervisors/teachers. Lastly, witnesses to discriminatory treatment should be supported to go from silent and passive bystanders to allies that are trained to see, understand, support, and search for the best ways to act.

## Data Availability

The datasets (interview transcripts) generated and analyzed during the current study are not publicly available due to the sensitive nature of the data and due to reasons of confidentiality, but are available from the corresponding author on reasonable request.
